# Effects of Arbuscular Mycorrhiza on Primary Metabolites in Phloem Exudates of *Plantago major* and *Poa annua* and on a Generalist Aphid

**DOI:** 10.3390/ijms222313086

**Published:** 2021-12-03

**Authors:** Jana Stallmann, Rabea Schweiger

**Affiliations:** Department of Chemical Ecology, Bielefeld University, 33615 Bielefeld, Germany; jana.stallmann@uni-bielefeld.de

**Keywords:** amino acids, aphids, arbuscular mycorrhizal fungi, carbohydrates, metabolite profiling, *Myzus persicae*, organic acids, *Poa annua*, phloem, *Plantago major*

## Abstract

Arbuscular mycorrhiza (AM), i.e., the interaction of plants with arbuscular mycorrhizal fungi (AMF), often influences plant growth, physiology, and metabolism. Effects of AM on the metabolic composition of plant phloem sap may affect aphids. We investigated the impacts of AM on primary metabolites in phloem exudates of the plant species *Plantago major* and *Poa annua* and on the aphid *Myzus persicae*. Plants were grown without or with a generalist AMF species, leaf phloem exudates were collected, and primary metabolites were measured. Additionally, the performance of *M. persicae* on control and mycorrhizal plants of both species was assessed. While the plant species differed largely in the relative proportions of primary metabolites in their phloem exudates, metabolic effects of AM were less pronounced. Slightly higher proportions of sucrose and shifts in proportions of some amino acids in mycorrhizal plants indicated changes in phloem upload and resource allocation patterns within the plants. Aphids showed a higher performance on *P. annua* than on *P. major*. AM negatively affected the survival of aphids on *P. major*, whereas positive effects of AM were found on *P. annua* in a subsequent generation. Next to other factors, the metabolic composition of the phloem exudates may partly explain these findings.

## 1. Introduction

The roots of most terrestrial plant species are colonized by arbuscular mycorrhizal fungi (AMF). Via their extensive extraradical mycelia, these endosymbionts deliver water and nutrients (mainly phosphate, but also nitrogen-containing ones) to their host plants, while they get photoassimilates from their hosts [1,2,3]. Although the AMF are restricted to the roots of the plants, several systemic effects of arbuscular mycorrhiza (AM) are known. Many studies reported positive effects of AM on aboveground plant growth, but neutral or negative effects have also been shown [4]. Plant photosynthetic CO_2_ assimilation is often enhanced in mycorrhizal compared to non-mycorrhizal plants [5]. Moreover, various effects of AM on the leaf metabolome, including primary and specialized (=secondary) metabolites, are known [6]. AM can also affect fruit quality, as shown in red tomato fruits with higher amino acid concentrations under AM conditions [7]. Interactions of plants with their antagonists are affected by AM as well. Effects on plant-herbivore interactions are highly variable, depending on the AMF species as well as on the feeding mode (i.e., chewers, phloem feeders, and mesophyll feeders) of the herbivore and its degree of feeding specialization [8]. Phloem-feeding aphids are often positively affected by AM [8,9]. Such effects may at least be partly mediated by AM effects on the metabolic composition of plant phloem sap. However, although there are several studies addressing impacts of AM on the foliar metabolome across plant species [6], studies targeting AM effects on plant phloem sap are rare (but see, for example, Pons et al. [10]). In particular, it is unclear whether such effects of AM on plant phloem sap are plant species-specific and whether this translates into plant species-specific effects on aphids.

The metabolic composition of the phloem sap of plants plays a decisive role in AMF–plant–aphid interactions. The phloem sieve tubes, which contain the phloem sap, are important transport routes for various metabolites within plants, connecting source and sink tissues/organs [11,12]. Photoassimilates that are produced in photosynthetically active parts of the plant shoots are uploaded into the phloem sieve tubes and distributed within the plants. Plant roots and the AMF colonizing them are sinks for photoassimilates. Indeed, the positive effects of AM on plant photosynthesis are, next to the AMF-facilitated nutrient uptake, due to the carbon sink strength imposed by the AMF [5]. Specifically, sucrose is transported via the phloem sap from aboveground plant parts to the roots, where it is unloaded and cleaved to fructose and glucose, with the latter monosaccharide probably being the main form of carbohydrate that is then delivered to the AMF [1,13,14]. As the carbon sink strength and resource allocation patterns within plants probably depend on the developmental stage of the AMF–plant interaction, studies including different developmental stages are needed to get mechanistic insights into AM effects on the plant phloem sap metabolome.

Moreover, the metabolic composition of the phloem sap and AM effects on this may impact plant–aphid interactions. Aphids are herbivores that specifically feed on the phloem sap of their host plants and can be, largely due to their parthenogenetic reproduction and short generation times, tremendous pests to plants [15]. For aphids, phloem sap is a challenging diet, because high sucrose concentrations bring along osmotic challenges, while amino acids (especially the essential ones) often occur in limiting concentrations [16]. Aphids show several adaptations to the consumption of phloem sap, such as the formation of oligosaccharides and their excretion via the honeydew, harboring endosymbionts, and the intermittent consumption of plant xylem sap [16,17,18]. Studies with artificial diets and plants revealed that aphid performance is influenced by the metabolic composition of their diet [19,20,21].

Given the crucial role of the plant phloem sap for tripartite AMF–plant–aphid interactions, the aim of the current study was to investigate the effects of AM on the metabolic composition of phloem exudates and on aphids across two plant species. Although specialized metabolites in the phloem sap may also play a role in plant–aphid interactions, we focused on primary metabolites in the current study. We applied an eco-metabolomics metabolite profiling approach [22], targeting carbohydrates, the sugar alcohol sorbitol, the cyclic polyol *myo*-inositol, organic acids of the tricarboxylic acid cycle, and amino acids. Specifically, we (i) assessed the AM effects on phloem exudates of *Plantago major* (Plantaginaceae) at different time points along the AMF–plant development, (ii) compared the AM effects on phloem exudates across the plant species *P. major* and *Poa annua* (Poaceae), and (iii) explored implications for the performance of the feeding generalist aphid species *Myzus persicae* (Hemiptera: Aphididae) on the two plant species. The two plant species were chosen to compare a dicotyledonous and a monocotyledonous species, for which the AM effects on the leaf metabolome are already known [23], while the AM effects on the metabolic composition of their phloem exudates have, to our knowledge, not been reported yet. We highlight that plant species identity is an important component that should be considered in AMF–plant–aphid interactions.

## 2. Results

Three different experiments were performed, each including control (NM, non-mycorrhizal) and AM (arbuscular mycorrhizal, i.e., inoculated with *Rhizoglomus irregulare*) plants. *Experiment I* (*Time Series*) focused on the influence of AM on *Plantago major* (Plantaginaceae), including three time points along the development of the AMF–plant interaction, i.e., T1 corresponding to 20 days post inoculation (dpi), T2 (30 dpi), and T3 (63 dpi). In *Experiment II* (*Species Comparison*), the AM effects on the two plant species *P. major* and *Poa annua* (Poaceae) were assessed at a late time point of the AMF–plant interaction (i.e., 64–68 dpi). In both experiments, the metabolic composition of the plant phloem exudates was assessed. The aim of *Experiment III* (*Aphid Performance*) was to investigate the effects of AM on the plants (61 dpi) and on a generalist aphid species (*Myzus persicae*; Hemiptera: Aphididae) on both plant species (*P. major*, *P. annua*) from 63 dpi onwards.

### 2.1. Root Colonization by AMF and Morphological Parameters

**Figure 1:**
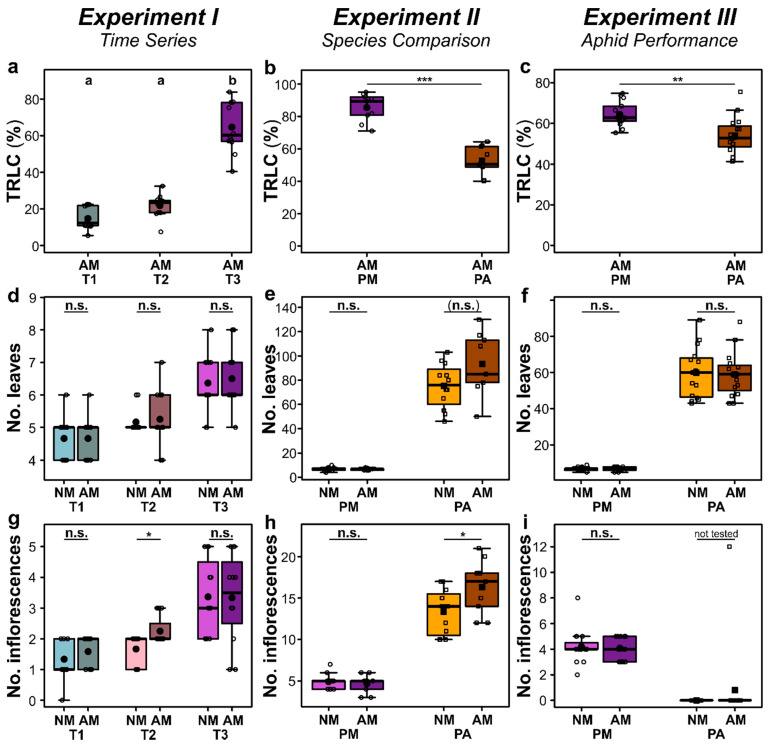
Total root length colonization (TRLC, first row), number of leaves (second row), and number of inflorescences (third row) of *Plantago major* (PM) and *Poa annua* (PA) plants that that have been left as controls (NM) or were inoculated with the arbuscular mycorrhizal fungus *Rhizoglomus irregulare* (AM). (**a**,**d**,**g**) *Experiment I* (*Time Series*) with *P. major*, showing data at the time points T1, corresponding to 20 days post inoculation (dpi), T2 (30 dpi), and T3 (63 dpi); (**b**,**e**,**h**) *Experiment II* (*Species Comparison*) with *P. major* and *P. annua* at 66–68 dpi (TRLC) and 64 dpi (numbers leaves/inflorescences); (**c**,**f**,**i**) *Experiment III* (*Aphid Performance*) with *P. major* and *P. annua*, showing TRLC at time points when all aphids had died on the individual plants or when the experiment was terminated, and numbers of leaves/inflorescences at 61 dpi (i.e., shortly before the aphid bioassay started). Data are given as box-whisker plots, with the medians given as horizontal thick lines, means as large filled symbols (circles: *P. major*; squares: *P. annua*), interquartile ranges (IQR) as boxes, raw data points as small open symbols (circles: *P. major*; squares: *P. annua*), and the whiskers extending to the most extreme data points within maximum 1.5 times the IQR. At the top of each subpanel, results of statistical tests are given: (**a**) different letters indicate significant differences of a post hoc Kruskal–mc test at *p* < 0.05, (**b**–**i**) asterisks indicate significant differences between groups [n.s., not significant; (n.s.) *p* < 0.1; *, *p* < 0.05; **, *p* < 0.01; ***, *p* < 0.001; (**b**,**c**,**f**) *t*-tests, (**d**,**e**,**g**–**i**) Mann–Whitney *U*-tests]. The sample sizes (biological replicates) were: *n* = 11–12 (*Experiment I*), *n* = 9–12 (*Experiment II*), *n* = 14–15 (*Experiment III*).

The AMF colonization of *P. major* roots, expressed as total root length colonization (TRLC), significantly differed between time points (Kruskal–Wallis test: *Χ*^2^ = 26.5, df = 2, *p* < 0.001) and increased over time, being significantly higher at 63 dpi (T3) than at 20 dpi (T1) and 30 dpi (T2) (*Experiment I*; Figure 1a). Roots of *P. major* showed generally significantly higher TRLC than those of *P. annua* (*Experiments II*, *III*; Figure 1b,c). The numbers of leaves and inflorescences of *P. major* increased over time (*Experiment I*; Figure 1d,g). *Poa annua* had generally more leaves than *P. major* (Figure 1e,f) and produced more inflorescences than *P. major* in *Experiment II*, while only one *P. annua* AM plant but all *P. major* plants flowered in *Experiment III* (Figure 1h,i). Although the numbers of leaves and inflorescences were not affected by the AM treatment in most cases, some positive effects of AM were found (Figure 1d–i). For *P. major*, the number of inflorescences was higher in AM than in NM plants at 30 dpi (T2, *Experiment I*). For *P. annua*, in *Experiment II* the number of leaves was slightly (marginally significant at *p* < 0.1) higher, while the number of inflorescences was significantly higher for AM than for NM plants.

### 2.2. Metabolic Composition of Phloem Exudates

**Figure 2:**
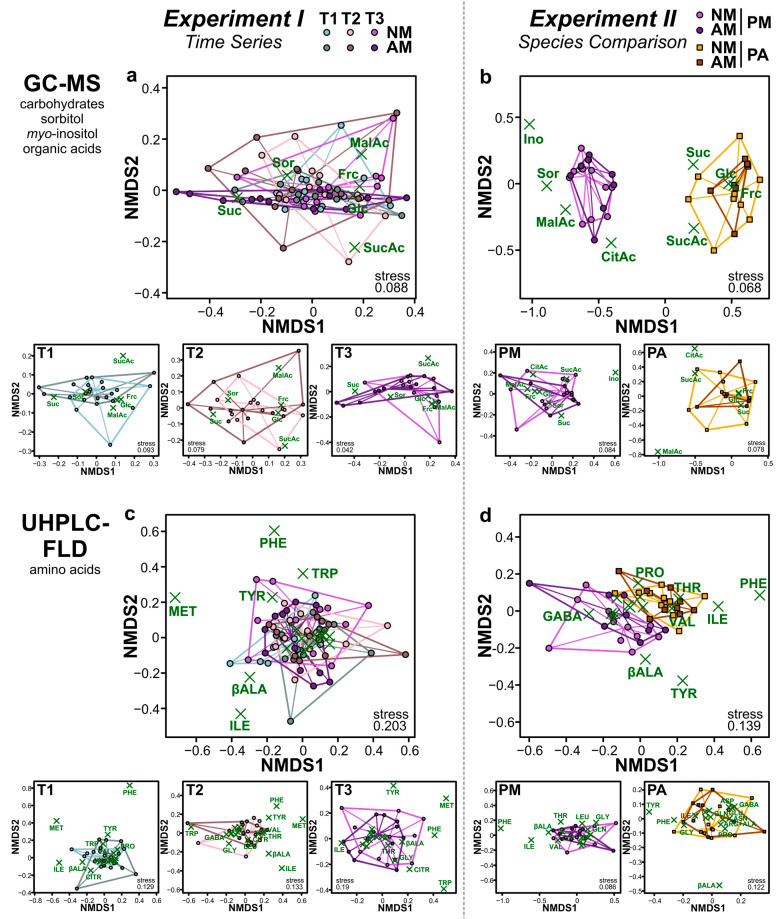
Non-metric multidimensional scaling (NMDS) plots showing the relative composition of primary metabolites (based on relative proportions) in phloem exudates of *Plantago major* (PM) and *Poa annua* (PA) plants that have been left as controls (NM) or were inoculated with the arbuscular mycorrhizal fungus *Rhizoglomus irregulare* (AM). Metabolites were measured per GC-MS (first row) and UHPLC-FLD (second row). (**a**,**c**) *Experiment I* (*Time Series*) with *P. major*, showing data at the time points T1, corresponding to 20 days post inoculation (dpi), T2 (30 dpi), and T3 (63 dpi), across all time points (top) and separately for each time point (bottom); (**b**,**d**) *Experiment II* (*Species Comparison*) with *P. major* and *P. annua* at 66–68 dpi, across both species (top) and separately for each species (bottom). Stress values of NMDS analyses are shown at the bottom. Different groups are surrounded by convex hulls, i.e., closed curves surrounding all data points, and all data points are connected to the corresponding medians of the treatment groups. Green crosses indicate loadings of the metabolites; in crowded regions of the plots, labels of metabolites with short loadings were partly omitted. Full names of the metabolites are given in Table 1. The sample sizes (biological replicates) were: *n* = 11–12 (*Experiment I*), *n* = 9–12 (*Experiment II*).

**Figure 3:**
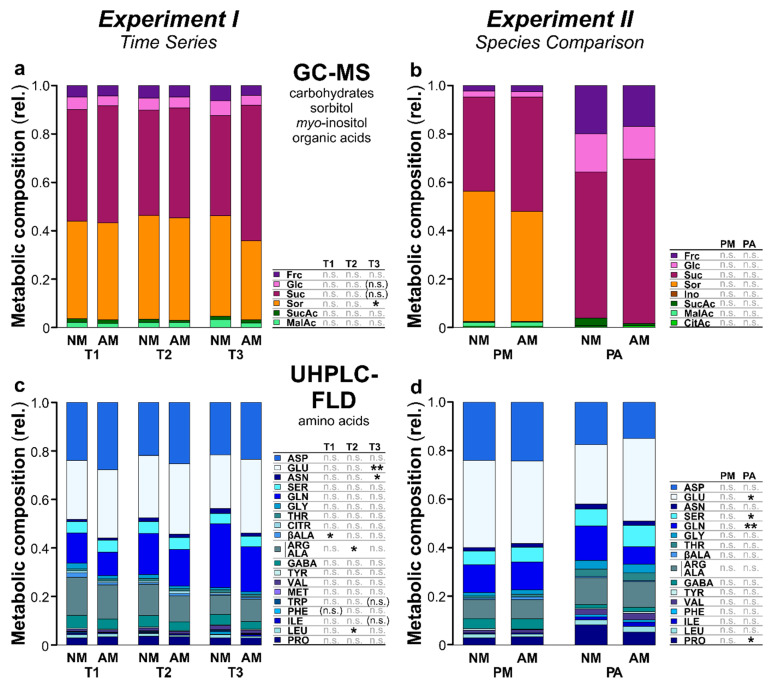
Relative composition of primary metabolites in phloem exudates of *Plantago major* (PM) and *Poa annua* (PA) plants that have been left as controls (NM) or were inoculated with the arbuscular mycorrhizal fungus *Rhizoglomus irregulare* (AM). Metabolites were measured per GC-MS (first row) and UHPLC-FLD (second row). (**a**,**c**) *Experiment I* (*Time Series*) with *P. major*, showing data at the time points T1, corresponding to 20 days post inoculation (dpi), T2 (30 dpi), and T3 (63 dpi); (**b**,**d**) *Experiment II* (*Species Comparison*) with *P. major* and *P. annua* at 66–68 dpi. Data are given as stacked bar plots representing mean metabolite proportions for each treatment group. Metabolites of the same class are shown in similar colors: carbohydrates in purple, the sugar alcohol sorbitol in orange, the cyclic polyol *myo*-inositol in brown, organic acids in green, and amino acids in blue. Full names of the metabolites are given in Table 1. For each time point (*Experiment I*) and plant species (*Experiment II*), results of pairwise Mann–Whitney *U*-tests comparing NM and AM plants within time points and species, respectively, are given [n.s., not significant; (n.s.) *p* < 0.1; *, *p* < 0.05; **, *p* < 0.01]. The sample sizes (biological replicates) were: *n* = 11–12 (*Experiment I*), *n* = 9–12 (*Experiment II*).

Plant phloem exudates were collected using the ethylenediaminetetraacetic acid (EDTA)-facilitated exudation technique [24]. In total, 28 primary metabolites were identified in the phloem exudates of *P. major* and/or *P. annua* via gas chromatography coupled to mass spectrometry (GC-MS) and ultra-high performance liquid chromatography coupled to fluorescence detection (UHPLC-FLD); arginine and alanine were chromatographically not resolved and thus quantified together (*Experiments I*, *II*; Table 1). Next to carbohydrates (two monosaccharides and the disaccharide sucrose), the sugar alcohol sorbitol, the cyclic polyol *myo*-inositol, three organic acids of the tricarboxylic acid cycle, and several amino acids (most of them being primary amino acids, except for the secondary amino acid proline) were found. Metabolite profiles of the phloem exudates were compared between samples of the different treatment groups, using relative proportions (0–1 scale) of the metabolites and non-metric multidimensional scaling (NMDS). There were only minor differences in the metabolite profiles of the phloem exudates of *P. major* between time points (*Experiment I*; Figure 2a,c). In contrast, in *Experiment II* the two plant species pronouncedly differed in their phloem exudate composition, both qualitatively and quantitatively (Table 1 and Figure 2b,d). While most metabolites that were found in the phloem exudates of *P. major* in *Experiment II* were also present in the phloem exudates of *P. annua*, sorbitol and *myo*-inositol were only found in *P. major* but not in *P. annua*. In addition to these qualitative differences between the plant species, several quantitative metabolic differences in terms of relative proportions of the metabolites were found (Figure 3b,d). The effects of the AM treatment on the metabolite profiles of the phloem exudates were minor (Figure 2). However, the relative proportions of some of the metabolites were affected by AM (Figure 3). In *Experiment I*, the relative proportions of sorbitol and glucose were significantly and by trend (marginally significant at *p* < 0.1), respectively, lower in AM than in NM *P. major* plants, while the proportion of sucrose was by trend (*p* < 0.1) higher in the AM plants (Figure 3a). Additionally, several amino acids differed in their relative proportions between NM and AM plants (Figure 3c). In *Experiment II* (Figure 3b,d), there were no significant differences for *P. major* metabolites between AM and NM plants, although similar (but not significant) effects as in *Experiment I* on sorbitol (lower in AM group) and sucrose (higher in AM group, also for *P. annua*) were seen. The proportions of several amino acids in phloem exudates of *P. annua* were affected by the AM treatment (Figure 3d). The sucrose-to-amino acid ratio was generally much (i.e., 13-fold based on means both in AM and in NM plants) lower in *P. annua* than in *P. major*. Moreover, it was higher in AM than in NM plants at all three time points of *Experiment I* (*P. major*; 1.4-fold higher mean in AM than in NM plants at T1; 2.0-fold at T2; 5.5-fold at T3) and for both plant species in *Experiment II* (1.1-fold higher in AM than NM plants for both species); the differences in the sucrose-to-amino acid ratios between AM and NM plants within time points and plant species were not significant, except for *P. major* at T3 (*Experiment I*) where a marginal significance (*p* < 0.1) was found (Mann–Whitney *U*-tests for *Experiment I*, *t*-tests for *Experiment II*).

### 2.3. Aphid Performance on Plants

**Figure 4:**
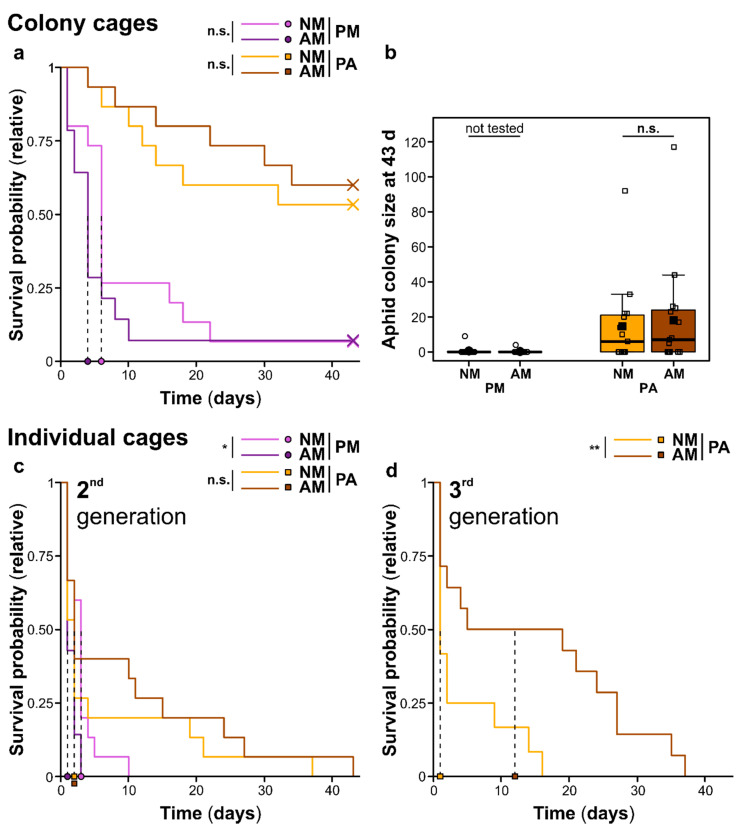
Performance of *Myzus persicae* aphids on *Plantago major* (PM) and *Poa annua* (PA) plants that have been left as controls (NM) or were inoculated with the arbuscular mycorrhizal fungus *Rhizoglomus irregulare* (AM) (*Experiment III*, *Aphid Performance*) in “colony cages” (first row) and in “individual cages” (second row). (**a**,**b**) Performance of aphid colonies, which started with five apterous adults each, i.e., (**a**) the survival of the colonies over time as Kaplan–Meier curves, with crosses indicating right censoring when the experiment was terminated and vertical dashed lines and symbols at the *x* axis indicating 50% survival, and (**b**) colony sizes 43 days after start of the aphid bioassay, as box-whisker plots with the medians given as horizontal thick lines, means as large filled symbols, interquartile ranges (IQR) as boxes, raw data points as small open symbols, and the whiskers extending to the most extreme data points within maximum 1.5 times the IQR; (**c**,**d**) Survival of individuals in the second (**c**) and third ((**d**); for *P. annua* only) generation over time as Kaplan–Meier curves, with vertical dashed lines and symbols at the *x* axis indicating 50% survival. At the top of each subpanel, results of pairwise statistical tests ((**a**,**c**,**d**): log-rank tests; **b**: Mann–Whitney *U*-test) comparing NM and AM plants within species are given [n.s., not significant; *, *p* < 0.05; **, *p* < 0.01]. The sample sizes (biological replicates) were *n* = 14–15, except for the 3rd generation in the individual cages, where samples sizes were *n* = 12 (NM) and *n* = 14 (AM).

To assess the performance of the feeding generalist aphid species *Myzus persicae* on *P. major* and *P. annua* NM and AM plants, the survival and final sizes of aphid colonies, which started with five apterous (i.e., unwinged) adult aphids each, “in colony cages” as well as the survival of individual aphids in “individual cages” were monitored. The performance of *M. persicae* aphids was generally higher on *P. annua* than on *P. major* (Figure 4). At the end of the experiment (i.e., 43 days after start of the aphid bioassay), more than half of the aphid colonies in “colony cages” were still alive on *P. annua* (both for NM and AM plants), reaching colony sizes of up to 117 aphids, while on *P. major* the colonies died much earlier and only two colonies (one on a NM, one on an AM plant) survived until day 43 (Figure 4a,b). Likewise, the survival of individual aphids in “individual cages” was much higher on *P. annua* than on *P. major* (Figure 4c,d) and more nymphs were produced in these cages on *P. annua* than on *P. major* (data not shown). The effects of the AM treatment on *M. persicae* differed between the plant species. On *P. major*, effects of AM were rather negative, further lowering the already bad aphid performance on this plant species. Specifically, the survival of aphid colonies was slightly (not significantly) lower on AM than on NM *P. major* plants especially during the first half of the experiment (Figure 4a), while the survival of individual aphids was significantly lower on AM than on NM plants of this species (Figure 4c). In contrast, effects of AM on *M. persicae* on *P. annua* were rather positive. While the survival of aphid colonies, aphid colony sizes at day 43, and the survival of individual aphids of the second generation were only slightly (not significantly) higher on AM than on NM *P. annua* plants (Figure 4a–c), individual aphids of the third generation survived significantly better on AM than on NM plants of this species (Figure 4d). Likewise, more nymphs were produced by the individual aphids on *P. annua* AM than on NM plants. In the cages of the second generation, 54 nymphs were produced by six individuals on AM plants, while there were only 37 nymphs from three individuals on NM plants. In the third generation, seven aphids reproduced on AM plants giving birth to 69 nymphs, while on NM plants only four nymphs were produced by two individuals.

## 3. Discussion

Our study revealed that the effects of AM on plants not only depend on the developmental stage of the AMF–plant interaction, but also on the plant species. Moreover, the AM effects on plant–aphid interactions were plant species-specific.

The finding that the AMF root colonization of *P. major* was higher than that of *P. annua* fits to an earlier study on these two plant species and the same AMF species [23]. Indeed, grasses often show lower colonization by AMF than dicotyledonous forbs [25,26]. This is probably related to the finer roots system of the grasses, because the structure of the root system is related to the nutrient acquisition strategy and is related to the probability to interact with AMF [26,27]. The numbers of leaves and inflorescences were not affected by AM, except for positive effects on the number of inflorescences of *P. major* at T2 (*Experiment I*) and of *P. annua* in *Experiment II*. Effects of AM on plant biomass-related traits are often positive, but neutral or negative effects were also reported [4]. Usually, forbs show stronger mycorrhizal growth responses than grasses [26,28]. The general low responsiveness in terms of growth-related parameters to the AM treatment found in the current is consistent with an earlier study, where the aboveground biomass of both species was unaffected by AM [23], and indicates that nutritional conditions in NM pots were not or were only slightly limiting. Importantly, the lack of strong effects on the numbers of leaves and inflorescences in our study allowed us to assess the effects of AM on the metabolic composition of leaf phloem exudates and on aphids with no/weak confounding effects by AM effects on growth-related traits. 

Several primary metabolites of different compound classes were detected in the phloem exudates of *P. major* and *P. annua* in the current study. Whether glucose is an artifact of the ethylenediaminetetraacetic acid (EDTA)-facilitated exudation technique [24] that was applied to collect phloem exudates is unclear; while some plant species seem to transport hexoses in the phloem sap, others do not, as discussed by van Bel and Hess [29]. The repertoire of primary metabolites we found largely fits to the metabolites reported for the leaves of these species [23]. Sorbitol, which was only found in leaves [23] and phloem exudates (current study) of *P. major* but not in those of *P. annua*, is, next to sucrose, a major component of the phloem sap of plantain species [30,31]. Although absolute concentrations of metabolites in phloem exudates cannot be assessed with the EDTA-facilitated exudation technique, the differences in the relative proportions of metabolites between the plant species indicate that their phloem exudates differ not only qualitatively, but also quantitatively. Large quantitative differences in several primary metabolites were also found in leaves of these two species [23]. The finding that the sucrose-to-amino acid ratio of the phloem exudates differed between the species fits to a study by Wilkinson and Douglas, who showed that sucrose-to-amino acid ratios in phloem exudates varied across several plant species [32].

There were only slight AM effects on the relative metabolic composition of the phloem exudates compared to the large differences between the plant species. Similarly, Pons et al. [10] showed that effects of AM on wheat (Poaceae) phloem exudates were minor, while the effects of drought stress were pronounced. However, this does not rule out that absolute concentrations, which cannot be assessed when using the EDTA-based phloem exudate collection method, were affected by AM. At the leaf level, several effects of AM on primary metabolites were reported in various plant species [6], with effects on organic acids of the tricarboxylic acid cycle differing between dicotyledonous species (including *P. major*) and the monocot *P. annua* [23]. The slightly higher proportions of sucrose in the phloem exudates of AM compared to NM plants of both plant species in the current study, accompanied by lower proportions of sorbitol in *P. major*, probably indicate an intensified and preferential upload of sucrose into the phloem sap in mycorrhizal plants. Indeed, it is known that large proportions (up to 20%) of the photoassimilates of plants are transported to the belowground AMF [33]. Specifically, sucrose is loaded into phloem sieve tubes in aboveground source tissues, transported to the roots, unloaded, and cleaved to fructose and glucose, with the latter one probably being the main form of carbohydrates delivered to the AMF [1,13,14]. The higher photosynthesis rates that are often observed in AM compared to NM plants [5], including *P. major* [34], probably contribute to the capability of plants to deliver enough photoassimilates to their fungal partners. The fact that in *Experiment I* these effects were only seen at the last time point indicates that a well-developed AMF–plant interaction is needed for changes in preferential phloem-loading processes. Likewise, metabolic effects of AM on the leaf metabolome of *P. major* increased along with the development of the AMF–plant interaction, probably related to shifts in the costs/benefits for the plant and resource allocation patterns [34]. Next to the shifts in the proportions of carbohydrates in the phloem exudates, the proportions of several amino acids were affected by AM, probably related to changes in nutrition and/or resource allocation.

The better performance of *M. persicae* aphids on *P. annua* than on *P. major* may be due to the much lower sucrose-to-amino acid ratios in the phloem exudates of the grass species. In general, for aphids, sucrose occurs in excess concentrations in the phloem sap, while amino acids are often limiting [16]. Although *M. persicae* is quite tolerant regarding the sucrose-to-amino acid ratio of its diet within certain limits, as shown with artificial diets [35], the huge differences in the sucrose-to-amino acid ratios between the plant species in our study are probably outside of its tolerance range. The absolute concentrations of the metabolites, which could not be assessed in the current study, probably also play a role. In addition, other metabolites not occurring in the phloem exudates but in the leaves of the two plant species [23], including specialized metabolites, as well as anatomical/morphological differences between the plant species may have influenced the acceptance of the host plants by the aphids and their performance.

While aphids are often positively affected by mycorrhization of their host plants [8,9], we found that AM effects on *M. persicae* were rather negative on *P. major*, whereas aphids were positively affected by AM on *P. annua*. This is in contrast to studies where positive effects of AM on *M. persicae* on *Plantago lanceolata* (Plantaginaceae) [36,37] and negative effects of AM on *Rhopalosiphum padi* (Hempitera: Aphididae) on *Phleum pratense* (Poaceae) have been reported [38], indicating that effects are highly dependent on the investigated study system and the traits measured. AM effects on aphids may also depend on the developmental stage of the AMF-plant interaction and/or on plant age [39]. Whether the slightly higher sucrose-to-amino acid ratios found in phloem exudates of *P. major* at T3 (*Experiment I*) (but not in *Experiment II*) or other metabolic changes further lowered the already bad aphid performance on *P. major* cannot be answered. Similarly, we cannot uncover whether AM-induced metabolic changes in the phloem sap contributed to the positive AM effects on the aphids on *P. annua*. AM effects on specialized metabolites may also play a role for plant–aphid interactions. For example, foliar concentrations of the iridoid glycoside aucubin were not or were positively affected by AM in other studies on plantain species [23,40,41]. In addition, volatile organic compounds play a role in AMF–plant–aphid interactions [42]. Further studies are needed where absolute concentrations of metabolites in the phloem sap are measured within the same experiment and at several time points along the aphid bioassay, in order to link the phloem exudate metabolome with aphid performance. Moreover, specialized metabolites should be targeted in addition to link nutritional effects with defensive properties of the host plants.

In general, there are several mechanisms that may contribute to the AM-induced metabolic changes in plants and effects on aphids, including the AMF-mediated improved nutrition of the plants, the carbon sink activity of the fungal endosymbiont, and the AM effects on plant signaling pathways as well as on plant anatomy/morphology. Interestingly, AM effects on the leaf metabolome of *P. major* were largely not phosphorus-mediated [34] and the positive effects of AM on the volatile-based attractiveness of *Vicia faba* (Fabaceae) to *Acyrthosiphon pisum* (Hemiptera: Aphididae) aphids seem to be mediated by effects on plant signaling, rather than by increased phosphorus supply [43]. For AM effects on photosynthesis, the carbon sink strength of the AMF plays a major role next to the improved plant nutrition [5]. Furthermore, AM effects on the carbon contents or thickness of the leaves and on vascular bundle sizes may contribute to AM effects on aphids by modulating the accessibility of the phloem sieve tubes for these herbivores [44,45]. To understand whether differences in the acceptance of host plants contribute to the variation in aphid performance on the different plant species and on NM versus AM plants, electrical penetration graph (EPG) studies targeting aphid feeding behavior should be performed in future. 

In conclusion, our study highlights the complexity of AMF–plant–aphid interactions and the importance of specifically assessing metabolic traits of plant phloem sap to better understand these ecological interactions. Adding to the study by Koricheva et al. [8], who revealed that the AM effects on herbivores depend on many other factors, we show that the identity of the host plant species is also highly relevant. Moreover, next to plant-mediated AM effects on aphids that feed aboveground, aphids may also affect the belowground AMF by reducing the plant carbon allocation to the endosymbionts [46]. Moreover, other plant individuals may be affected by AMF–plant–aphid interactions due to connections via common mycorrhizal/mycelial networks [47]. Thus, future studies should address AMF–plant–aphid interactions from various perspectives.

## 4. Materials and Methods

In total, three experiments were performed. All experiments included non-mycorrhizal (NM) and mycorrhizal (AM) plants. The first two experiments focused on AM effects on the metabolic composition of phloem exudates, while in the third experiment the effects of AM on aphids were addressed. In *Experiment I* (*Time Series*), the effects of AM on *Plantago major* were investigated, including three time points: 20 days post inoculation (dpi) (T1), 30 dpi (T2), and 63 dpi (T3). In *Experiment II* (*Species Comparison*), the AM effects on *P. major* and on *Poa annua* were compared at a late time point (64–68 dpi). In *Experiment III* (*Aphid Performance*), effects on both plant species (61 dpi) and on the aphid species *Myzus persicae* (from 63 dpi onwards) were assessed.

### 4.1. Plant Cultivation and Inoculation with AMF

The cultivation of plants and inoculation with AMF were done similar to Schweiger et al. [23]. The dicotyledonous species *Plantago major* L. (Plantaginaceae; PM) and the monocotyledonous species *Poa annua* L. (Poaceae; PA) were used to compare two functional types for which AM effects on primary and specialized metabolites in the leaves have already been described [23]. Plants were grown from seeds (PM: Blauetikett Bornträger, Offstein, Germany; PA: Appels Wilde Samen, Darmstadt, Germany), which were surface-sterilized with 70% ethanol (1 min) and 2% sodium hypochlorite (10 min), and swelled in demineralized water for 2 h. Then, seeds of both plant species were separately put into a sterile (121 °C, 20 min) 3:2 (*v*:*v*) mixture of swelling clay (pH 9.6, 0.9 g L^−1^ salt content; Fibo ExClay, Lamstedt, Germany), which had been washed to reduce the nutrient concentrations before, and sand (0.1–0.5 mm particle size; Quarzwerke, Frechen, Germany). After 3 days at 4 °C in the dark, the substrate with seeds was transferred to a climate chamber (20 °C; 60–70% relative humidity; 16:8 h light:dark; mean daytime light conditions at pot height: UV-A 0.761 W m^−2^, UV-B 0.023 W m^−2^, photosynthetically active photon flux density 208 µmol m^−2^ s^−1^). Then, at 15 (*Experiments I*, *II*) or 18 (*Experiment III*) days after sowing, seedlings were piqued into small trays (pots with d = 5 cm). A total of 41 days after sowing, seedlings were transferred to 2 L pots (14 cm × 14 cm × 14.5 cm) containing the substrate described above. Within each experiment, half of the pots were randomly assigned to the AM treatment, i.e., plants were inoculated with the generalist AMF species *Rhizoglomus irregulare* (Błaszk., Wubet, Renker, and Buscot) Sieverd., G. A. Silva, and Oehl (2015) (Glomeromycotina), using 180 mL of a vermiculite-based (*Experiments I*, *II*) and 200 mL of a sand-based (*Experiment III*) inoculum from Inoq (Schnega, Germany) per pot. This fungal species was chosen, because as a generalist fungus it colonizes different plant species including *P. major* and *P. annua* [23], allowing comparisons of the two plant species included in our study. NM plants received the same amount of sterilized (121 °C, 20 min) inoculum instead. Before the inoculum was sterilized for these plants, a microbial wash was prepared from the inoculum using demineralized water. This microbial wash was filtered (20 µm sieve; Retsch, Haan, Germany), thus containing small microorganisms from the inoculum but no *R. irregulare* spores. The filtrate was applied to the NM pots; the AM plants received the same amount of demineralized water instead. Pots were covered with a layer (circa 1 cm) of sterile sand. For all experiments, plants of different treatments (i.e., different harvest dates, different species, NM, and AM plants) were placed in randomized block designs. Plants were watered with tap water several times per week as needed with the same water volume per pot. In addition, plants were regularly fertilized with a Hoagland solution [48], which was modified according to Schweiger et al. [23]. The concentrations of KH_2_PO_4_ in the fertilizer solution were reduced after AM plants had been inoculated with the AMF species for all plants to create phosphate-poor conditions facilitating mycorrhizal colonization. The amounts of fertilizer were increased along with the growth of the plants, with all pots receiving the same volume of fertilizer at each time point; during the aphid bioassay (*Experiment III*, from 63 dpi onwards), plants were not fertilized anymore. The numbers of leaves and inflorescences of the plants were assessed. In *Experiment I*, this was done at the time points when phloem exudates were collected (i.e., 20 dpi, 30 dpi, 63 dpi), while in *Experiment II* it was done at 64 dpi, i.e., shortly before phloem exudates were collected at 66–68 dpi. In *Experiment III*, these parameters were assessed at 61 dpi, i.e., shortly before the aphid bioassays started at 63 dpi. Original sample sizes were *n* = 12 (*Experiment I*), *n* = 12 (*Experiment II*) and *n* = 15 (*Experiment III*).

### 4.2. Collection of Phloem Exudates

Phloem exudates of *P. major* at different time points (*Experiment I*; 20 dpi, 30 dpi, 63 dpi) and of *P. major* and *P. annua* at 66–68 dpi (*Experiment II*) were collected in the afternoon hours using the ethylenediaminetetraacetic acid (EDTA)-facilitated exudation technique [24] modified after Jakobs and Müller [49]. Leaf material (*P. major*: youngest fully expanded leaf, the next younger and the next older one; *P. annua*: 10 young, fully expanded leaf blades) was cut at the bases of the petioles (*P. major*) or at the base of the leaf blades (*P. annua*), the cutting edges were incubated in 1 mL 8 mM EDTA (99%; AppliChem, Darmstadt, Germany) solution (adjusted to pH 7 with NaOH) at 20 °C in the dark for 2 h, washed with Millipore water, and incubated in Millipore water for further 2 h (dark, 20 °C). Then, the phloem exudate samples were immediately quenched in liquid nitrogen and stored at −80 °C until the metabolic analyses (see below). 

### 4.3. Metabolite Profiling

Profiling of primary metabolites in the phloem exudates was performed modified after Jakobs and Müller [49], using subsamples of the phloem exudates and two different analytical platforms.

For the analysis of carbohydrates, the sugar alcohol sorbitol, the cyclic polyol *myo*-inositol, and organic acids of the tricarboxylic acid cycle, 300 µL subsamples were lyophilized, redissolved in 200 µL (*Experiment I*) or 150 µL (*Experiment II*) 80% methanol (LC-MS grade; Fisher Scientific, Loughborough, UK) containing the internal standard ribitol (Sigma–Aldrich, Steinheim, Germany), 35 µL of the supernatants were dried under nitrogen, and derivatized at 37 °C under stirring via methoximation with 75 µL (*Experiment I*) or 50 µL (*Experiment II*) *O*-methylhydroxylamine hydrochloride (≥98%; Sigma–Aldrich) in pyridine (20 mg mL^−1^) for 90 min, followed by silylation with 75 µL (*Experiment I*) or 50 µL (*Experiment II*) *N*-methyl-*N*-trimethylsilyl-trifluoroacetamide (≥95%; Macherey-Nagel, Düren, Germany) for 30 min. Then, the samples were analyzed using gas-chromatography coupled to mass spectrometry (GC-MS; Focus GC-DSQII, Thermo Electron, Rodany, Italy), using a VF-5 ms column (30 m × 0.25 mm i.d., circa 10 m guard column; Varian, Palo Alto, CA, USA). The GC was operated using the following settings: injector temperature 225 °C, split 1:10, carrier gas (helium) flow 1.2 mL min^−1^, oven temperature 80 °C for 3 min followed by a ramp (5 °C min^−1^) to 325 °C. The transfer line temperature was 250 °C. The MS settings were: electron impact positive ionization at 70 eV, mass-to-charge (*m*/*z*) range 50 to 750, full scan mode. In addition to the plant samples, blank samples without plant material and n-alkanes (C8-C40; Sigma–Aldrich) were measured. Data analyses were done in XCalibur 1.4 SR1 (Thermo Electron). The internal standard ribitol was used to check the quality of the data and blanks were used to assess background contaminations. Based on the n-alkanes, retention indices (RI) according to Kováts 1958 [50] were calculated. Analytes were identified based on their RI and mass spectra by comparing these parameters with the entries in the Golm metabolome database (GMD) [51,52], the qualifier and quantifier *m*/*z* listed in the mass spectral and retention time (RT) index libraries [53], and by comparing with reference standards listed in an in-house database. Quantification via peak areas was done based on total ion chromatograms, including only peaks with a signal-to-noise ratio ≥ 3. Peak areas of analytes belonging to the same metabolite, occurring due to the derivatization procedure, were summed (i.e., two analytes for fructose and glucose each). Only metabolites that were present in at least half of the replicates of at least one treatment group (*Experiment I*: time point x mycorrhiza treatment; *Experiment II*: plant species x mycorrhiza treatment) were retained in the dataset. Because absolute concentrations of metabolites could not be determined due to unknown (and maybe treatment group-dependent) amounts of phloem sap entering the collection solution, data were used as relative proportions on a 0–1 scale. 

For amino acid profiling, 300 µL subsamples were lyophilized and redissolved in 80% (*v*:*v*) methanol (LC-MS grade; Th. Geyer, Höxter, Germany) with the internal standards norvaline and sarcosine (Agilent Technologies, Waldbronn, Germany). Samples were subjected to ultra-high performance liquid chromatography coupled to fluorescence detection (UHPLC-FLD; 1260/1290 Infinity; Agilent Technologies, Santa Clara, CA, USA). The autosampler was operated at 6 °C. In the autosampler needle, amino acids were derivatized by mixing and incubating with borate buffer (0.4 M, pH 10.2; Agilent Technologies), ortho-phthaldialdehyde (OPA; in borate buffer and 3-mercaptopropionic acid; Agilent Technologies), 9-fluorenyl-methyl chloroformate (FMOC; in acetonitrile; Agilent Technologies), and injection diluent, which was a mixture of 100 mL eluent A (see below) and 0.4 mL 85% phosphoric acid (AppliChem). Then, samples were injected and derivatized amino acids were separated on a ZORBAX Eclipse Plus C18 column (250 mm × 4.6 mm, 5 µm particle size, with guard column; Agilent Technologies) at 40 °C and a flow rate of 1.5 mL min^−1^. A gradient from eluent A [per 1 L Millipore water: 1.4 g Na_2_HPO_4_ (>99.5%; AppliChem), 3.8 g Na_2_B_4_O_7_ ∙ 10 H_2_O (≥99.5%; Sigma–Aldrich, St. Louis, MO, USA), and 32 mg NaN_3_ (≥98%; Carl Roth, Karlsruhe, Germany); adjusted to pH 8.2] to eluent B [4.5:4.5:1 (*v*:*v*:*v*) mixture of methanol (LC-MS grade; Fisher Scientific), acetonitrile (LC-MS grade; VWR International S.A.S., Fontenay-sous-Bois, France) and Millipore water] was applied: 2% B for 0.84 min, then linearly increased to 57% (reached at 33.4 min), followed by column cleaning and equilibration. OPA-derivatized primary amino acids were detected using an excitation wavelength of 340 nm and an emission wavelength of 450 nm; for FMOC-derivatized secondary amino acids, which eluted later, 260 nm and 325 nm were used as excitation and emission wavelengths, respectively. In addition to the plant samples, blank samples without plant material and mixtures of reference standards were measured. Data were analyzed in OpenLab ChemStation C.01.07 (Agilent Technologies). Data quality was checked via the internal standards norvaline and sarcosine and blanks were used to assess background contaminations. Amino acids were identified by comparing retention times with those of the reference standards and quantified via their peak heights. Arginine and alanine could not be chromatographically separated, forming a broad peak, and were thus quantified together. As for the metabolites measured via GC-MS (see above), only amino acids which occurred in at least half of the replicates of at least one treatment group were retained in the dataset and data were used as relative proportions on a 0–1 scale.

### 4.4. Aphid Bioassays

For *Experiment III* (*Aphid Performance*), *Myzus persicae* Sulzer (Hemiptera, Aphididae) aphids were used, because as a feeding generalist this species infests various host plant species [54], allowing comparisons of the two plant species included in our study. Aphids were originally taken from *Brassica rapa* L. ssp. *pekinensis* (Brassicaceae) plants in a greenhouse and reared for several generations at room temperature and a 16:8 h light:dark rhythm in tents with a mixture of *P. major* and *Plantago lanceolata* (Plantaginaceae; for bioassays on *P. major*) or with *P. annua* (for bioassays on *P. annua*). Under these conditions, only parthenogenetically reproducing viviparous females of *M. persicae* occur, similar to spring/summer seasons in temperature regions. The plants in the tents were regularly exchanged to avoid the production of alate (i.e., winged) aphids due to crowding. Apterous (i.e., unwinged) aphids were used for the experiment. Aphids were handled very carefully using a wettish brush.

Pots with *P. major* and *P. annua* plants were transferred from the climate chamber (see above) to another room (25 °C; 25% relative humidity; 16:8 h light:dark; mean daytime light conditions at pot height: UV-A 0.340 W m^−2^, UV-B 0.017 W m^−2^, photosynthetically active photon flux density 104 µmol m^−2^ s^−1^) shortly before the aphid bioassay started, keeping the randomized block design. The aphid bioassay was performed using clip cages with aphid colonies and individual aphids. At 63 dpi, five apterous *M. persicae* adults were put on the abaxial sides of the leaf tips of the youngest fully established leaves of each plant; for *P. annua*, several leaf blades were combined to get a larger leaf surface. Aphids were fixed on the leaves with clip cages (inner diameter 16 mm, height 15 mm) consisting of acrylic glass, gauze for ventilation, and foam rubber to avoid damage of the leaves. In these “colony cages”, nymphs produced within the first two days were removed, whereas nymphs produced from the third day onwards were left on the plants. Adults and nymphs that were alive were counted every other day for 43 days, while dead aphids were removed. Two days after setting up the “colony cages”, one additional “individual cage (2nd generation)” per plant was installed on the same leaf/leaves. One nymph from the corresponding “colony cage” on the same plant (or, if no nymph was available, from another replicate of the same treatment group or from the corresponding rearing tent) was put into each of these cages. The first nymphs produced by the individuals in the “individual cages (2nd generation)” within 8 days (i.e., the first day at which nymphs had been born) to 13 days were individually put into further “individual cages (3rd generation)” on the same leaves; if no nymph from the same plant was available, a nymph from another plant of the same treatment group was used. Thus, each plant was finally equipped with three clip cages. Empty clip cages (i.e., in which colonies or individuals had died) were left on the plants. On a daily basis, the survival and reproduction of the individuals in the “individual cages” were monitored and offspring and dead aphids were removed until all individuals in the “individual cages” had died. Because the survival and reproduction of individual aphids on *P. major* was very low, data for the 3rd generation are only shown for *P. annua*, for which *n* = 12 (NM) and *n* = 14 (AM) individuals were followed, corresponding to the number of nymphs produced on plants of these treatment groups between day 8 and 13 after onset of the individual cages (2nd generation). 

### 4.5. Determination of Intraradical Mycorrhization

To assess the colonization of plant roots with AMF, roots and substrate were separated and roots were washed. For *Experiments I* and *II*, this was done within few days after the phloem exudates had been collected from the plants, while for *Experiment III* this was done as soon as all aphids had died on the corresponding plant or (if aphids had survived until then on the plant) at the termination of the experiment. For determination of the intraradical mycorrhization, roots were cut into fragments, subsamples were bleached in 10% KOH (15 min, 95 °C), washed and acidified with 1% (*v*:*v*) HCl for 5 min at room temperature, and stained (48 h, room temperature) in a 1:40 (*v*:*v*) mixture of trypan blue (0.4%; Sigma–Aldrich) and a 2:1:2 (*v*:*v*:*v*) solution of lactic acid (90%), glycerine (98%), and Millipore water. Afterwards, stained roots were conserved in 4:2:1 (*v*:*v*:*v*) lactic acid (90%), glycerine (98%), and Millipore water at 4 °C in the dark. The total root length colonization (TRLC) was determined using the grid-line intersect method [55], counting hyphae, arbuscules, and vesicles in about 200 intersects per sample. Within each experiment, some NM plants were randomly chosen and checked; no AMF colonization was found in the corresponding roots, indicating that there was no transfer of AM between treatment groups. 

### 4.6. Statistical Analyses

Final sample sizes were slightly reduced compared to the original ones, because for some plants the AM treatment failed (i.e., no or very low root AMF colonization levels) and because there were technical issues with the GC-MS or UHPLC-FLD analyses of some samples, and only those replicates were retained in the datasets, for which all parameters could be assessed. The final sample sizes were *n* = 11 for the T3 NM group and *n* = 12 for the other treatment groups of *Experiment I*; *n* = 9 for the groups PM AM and PA AM, *n* = 11 for PM NM, and *n* = 12 for PA NM in *Experiment II*; *n* = 14 for PM AM, *n* = 15 for the other groups of *Experiment III*.

All statistical analyses were done in R 4.1.1 [56], using the packages *car* [57], *pgirmess* [58], *survival* [59,60], *survminer* [61], and *vegan* [62]. A significance threshold of α = 0.05 was used. Data were tested for normal distribution using Shapiro–Wilk tests and for homoscedasticity using Levene tests. The TRLC of AM plants was compared between time points (*Experiment I*) using Kruskal–Wallis tests followed by Kruskal–mc tests. For pairwise comparisons of the TRLC between species (*Experiments II*, *III*) and of other parameters between NM and AM plants within time points (*Experiment I*) or within plant species (*Experiments II*, *III*), Mann–Whitney *U*-tests or *t*-tests were used, applying the same test for all pairwise comparisons of a certain parameter or set of metabolites within an experiment. The metabolic composition of phloem exudates was compared between treatment groups using non-metric multidimensional scaling (NMDS) analyses of relative proportions of metabolites (0–1 scale, see above), separately for GC-MS and UHPLC-FLD data, both across and within time points (*Experiment I*) and across and within plant species (*Experiment II*). For the NMDS analyses, data were standardized using Wisconsin double standardization of square root-transformed data. Kulczynski distances were used. The survival probabilities of aphid colonies (“colony cages”) and individuals (“individual cages”; 2nd generation for both plant species, 3rd generation for *P. annua* only) were plotted as Kaplan–Meier curves and the survival on NM versus AM plants within plant species was compared using log-rank tests.

## Figures and Tables

**Table 1:** Primary metabolites identified in phloem exudates of *Plantago major* (PM) and/or *Poa annua* (PA).

Metabolite ^1^	Analytical Platform ^2^	Retention Parameter ^3^		*m*/*z*^4^	Identification ^5^		Occurrence ^6^	
					GMD	Std.	PM	PA
Carbohydrates								
Fructose (Frc)	GC-MS	RI	1862/1871	217, 277, 364, 335, 307	√	√	√ ^I,II^	√
Glucose (Glc)	GC-MS	RI	1886/1904	319, 229, 343, 305, 160	√	√	√ ^I,II^	√
Sucrose (Suc)	GC-MS	RI	2614	451, 361, 319, 157, 437	√	√	√ ^I,II^	√
Sugar alcohol								
Sorbitol (Sor)	GC-MS	RI	1923	307, 157, 217, 331, 319	√	√	√ ^I,II^	
Cyclic polyol								
*myo*-inositol (Ino)	GC-MS	RI	2077	265, 318, 191, 507, 305	√	√	√ ^II^	
Organic acids								
Succinic acid (SucAc)	GC-MS	RI	1312	172, 147, 262, 129, 247	√	√	√ ^I,II^	√
Malic acid (MalAc)	GC-MS	RI	1485	245, 335, 307, 217, 233	√	√	√ ^I, II^	√
Citric acid (CitAc)	GC-MS	RI	1812	375, 211, 183, 257, 273	√	√	√ ^II^	√
Amino acids								
Aspartic acid (ASP)	UHPLC-FLD	RT	3.2 min			√	√ ^I,II^	√
Glutamic acid (GLU)	UHPLC-FLD	RT	4.9 min			√	√ ^I,II^	√
Asparagine (ASN)	UHPLC-FLD	RT	8.3 min			√	√ ^I,II^	√
Serine (SER)	UHPLC-FLD	RT	8.8 min			√	√ ^I,II^	√
Glutamine (GLN)	UHPLC-FLD	RT	10.0 min			√	√ ^I,II^	√
Glycine (GLY)	UHPLC-FLD	RT	11.0 min			√	√ ^I,II^	√
Threonine (THR)	UHPLC-FLD	RT	11.3 min			√	√ ^I,II^	√
Citrulline (CITR)	UHPLC-FLD	RT	12.1 min			√	√ ^I^	
β-alanine (βALA)	UHPLC-FLD	RT	12.7 min			√	√ ^I,II^	√
Arginine+alanine (ARG+ALA)	UHPLC-FLD	RT	13.7 min			√	√ ^I,II^	√
γ-aminobutyric acid (GABA)	UHPLC-FLD	RT	14.3 min			√	√ ^I,II^	√
Tyrosine (TYR)	UHPLC-FLD	RT	16.3 min			√	√ ^I,II^	√
Valine (VAL)	UHPLC-FLD	RT	19.6 min			√	√ ^I,II^	√
Methionine (MET)	UHPLC-FLD	RT	20.1 min			√	√ ^I^	
Tryptophan (TRP)	UHPLC-FLD	RT	21.8 min			√	√ ^I^	
Phenylalanine (PHE)	UHPLC-FLD	RT	22.4 min			√	√ ^I,II^	√
Isoleucine (ILE)	UHPLC-FLD	RT	22.7 min			√	√ ^I,II^	√
Leucine (LEU)	UHPLC-FLD	RT	23.9 min			√	√ ^I,II^	√
Proline (PRO)	UHPLC-FLD	RT	31.5 min			√	√ ^I,II^	√

^1^ Metabolites are grouped according to metabolite classes; names of acids are given in protonated form; abbreviations of metabolites are given in parentheses. ^2^ Gas-chromatography coupled to mass spectrometry (GC-MS) or ultra-high performance liquid chromatography coupled to fluorescence detection (UHPLC-FLD). ^3^ Kováts retention index (RI) or retention time (RT). If two analytes per metabolite were found, both RI are given. ^4^ Characteristic mass-to-charge ratios (*m*/*z*). ^5^ It is indicated whether metabolites were identified using the Golm metabolome database (GMD; for metabolites measured per GC-MS only) and/or reference standards (Std.). ^6^ Occurrence of metabolites in phloem exudates of *P. major* (PM) and/or *P. annua* (PA). For *P. major*, it is additionally indicated whether metabolites were found in *Experiment I* and/or *Experiment II*.

## Data Availability

The raw data associated with this study are available from the corresponding author upon reasonable request.
